# TRAIL-induced programmed necrosis as a novel approach to eliminate tumor cells

**DOI:** 10.1186/1471-2407-14-74

**Published:** 2014-02-07

**Authors:** Susann Voigt, Stephan Philipp, Parvin Davarnia, Supandi Winoto-Morbach, Christian Röder, Christoph Arenz, Anna Trauzold, Dieter Kabelitz, Stefan Schütze, Holger Kalthoff, Dieter Adam

**Affiliations:** 1 Institut für Immunologie, Christian-Albrechts-Universität, Michaelisstrasse 5, 24105 Kiel, Germany; 2 Sektion für Molekulare Onkologie, Institut für Experimentelle Tumorforschung, Christian-Albrechts-Universität, Kiel, Germany; 3 Institut für Chemie der Humboldt-Universität, Berlin, Germany

**Keywords:** Programmed necrosis, TRAIL, TNF, Ceramide, Chemotherapy

## Abstract

**Background:**

The cytokine TRAIL represents one of the most promising candidates for the apoptotic elimination of tumor cells, either alone or in combination therapies. However, its efficacy is often limited by intrinsic or acquired resistance of tumor cells to apoptosis. Programmed necrosis is an alternative, molecularly distinct mode of programmed cell death that is elicited by TRAIL under conditions when the classical apoptosis machinery fails or is actively inhibited. The potential of TRAIL-induced programmed necrosis in tumor therapy is, however, almost completely uncharacterized. We therefore investigated its impact on a panel of tumor cell lines of wide-ranging origin.

**Methods:**

Cell death/viability was measured by flow cytometry/determination of intracellular ATP levels/crystal violet staining. Cell surface expression of TRAIL receptors was detected by flow cytometry, expression of proteins by Western blot. Ceramide levels were quantified by high-performance thin layer chromatography and densitometric analysis, clonogenic survival of cells was determined by crystal violet staining or by soft agarose cloning.

**Results:**

TRAIL-induced programmed necrosis killed eight out of 14 tumor cell lines. Clonogenic survival was reduced in all sensitive and even one resistant cell lines tested. TRAIL synergized with chemotherapeutics in killing tumor cell lines by programmed necrosis, enhancing their effect in eight out of 10 tested tumor cell lines and in 41 out of 80 chemotherapeutic/TRAIL combinations. Susceptibility/resistance of the investigated tumor cell lines to programmed necrosis seems to primarily depend on expression of the pro-necrotic kinase RIPK3 rather than the related kinase RIPK1 or cell surface expression of TRAIL receptors. Furthermore, interference with production of the lipid ceramide protected all tested tumor cell lines.

**Conclusions:**

Our study provides evidence that TRAIL-induced programmed necrosis represents a feasible approach for the elimination of tumor cells, and that this treatment may represent a promising new option for the future development of combination therapies. Our data also suggest that RIPK3 expression may serve as a potential predictive marker for the sensitivity of tumor cells to programmed necrosis and extend the previously established role of ceramide as a key mediator of death receptor-induced programmed necrosis (and thus as a potential target for future therapies) also to the tumor cell lines examined here.

## Background

Programmed cell death (PCD) is an important cellular mechanism whose dysregulation is involved in many human pathologies, especially tumor formation. Induction of PCD through the activation of caspases (apoptosis) is the best characterized route to death in most cell types [1]. Independent from apoptosis, programmed necrosis represents an alternative form of PCD that operates without detectable caspase activity [2,3]. Yet incompletely understood, the mechanisms of programmed necrosis need to be intensively investigated, because a better knowledge of these pathways may directly translate into improved therapies for cancers resistant to apoptosis [4,5]. Our own group has previously identified the sphingolipid ceramide as one of the pivotal mediators in death receptor-mediated programmed necrosis [3,6,7]. Although the induction of (caspase-dependent) apoptosis via manipulation of intracellular ceramide levels is increasingly recognized as an option in tumor therapy [8], detailed information on the induction of (caspase-independent) programmed necrosis by ceramide in clinically relevant tumor cell systems is currently unavailable. Programmed necrosis induced by tumor necrosis factor (TNF)-receptor 1 (TNF-R1) currently represents the most comprehensively studied system. Yet, the potential usefulness of TNF in clinical oncology is severely limited by its strong systemic toxic side effects. As an alternative, TNF-related apoptosis inducing ligand (TRAIL) can selectively induce apoptosis in tumor cells while leaving non-transformed cells mostly unaffected [9]. However, many tumor cells are intrinsically resistant against TRAIL-induced apoptosis and, even when combined with chemo- or radiotherapy, a resounding breakthrough in the therapy of cancer patients has not yet been achieved. As a potential alternative, we and others have previously demonstrated the ability of human and murine TRAIL receptors to induce programmed necrosis independently from their apoptotic capabilities when induction of apoptosis fails or is actively inhibited [7,10]. In consequence, the induction of programmed necrosis by TRAIL may represent a novel and additional, but still largely unexplored option for the elimination of tumor cells, in addition to the well-established strategies aimed at the induction of apoptosis.

In this study, we have therefore investigated the effects of TRAIL-induced programmed necrosis on a panel of 14 distinct human cancer cell lines of diverse origin (i.e. leukemia (U-937, CCRF-CEM), gall bladder adenocarcinoma (Mz-ChA-1), pancreatic adenocarcinoma (BxPC-3, Colo357, Panc89, PancTu-I, A818-4, Pt45P1), colorectal adenocarcinoma (HT-29), gastric adenocarcinoma (MKN-28), ovary adenocarcinoma (SK-OV-3), non-small cell lung carcinoma (KNS-62) and malignant melanoma (SK-Mel-28)). We show that TRAIL-induced programmed necrosis causes death of a wide range of these cell lines, impairs their clonogenic survival and acts in synergy with chemotherapeutic agents. Our findings also suggest that susceptibility/resistance of tumor cells to programmed necrosis is primarily determined by expression of the kinase RIPK3 (which indicates its potential usefulness as a predictive marker) and that ceramide represents a pivotal factor downstream of RIPK3 in the execution of programmed necrosis not only in the previously studied common laboratory cell lines, but also in the clinically more relevant tumor cell systems employed here.

## Methods

### Reagents

The Smac mimetic birinapant was provided by ChemieTek, Indianapolis, IN, USA. Necrostatin-1 and necrosulfonamide were obtained from Calbiochem, Darmstadt, Germany. Arc39 has been previously described [11,12]. Cisplatin, etoposide, trichostatin A, 5-fluorouracil, irinotecan, doxorubicin, camptothecin and paclitaxel were ordered from Sigma-Aldrich, Munich, Germany.

### Cell lines and culture conditions

Mz-ChA-1, Colo357, PancTu-I, Panc89, A818-4, Pt45P1, MKN-28 and KNS-62 cells have been described [13-16]. U-937, BxPC-3, HT-29, CCRF-CEM, SK-OV-3 and SK-MEL-28 cells were originally obtained from the American Type Culture collection. The identity of all cell lines was validated by STR profiling. The cell lines were cultured in RPMI 1640 (Life Technologies, Darmstadt, Germany) supplemented with 10% v/v FCS and 1 mM sodium pyruvate or (U-937 cells) 10% v/v FCS and 50 μg/ml penicillin/streptomycin. Wildtype and RIP3-deficient mouse embryonic fibroblasts (MEF) have been described [17] and were cultured in DMEM (Life Technologies) supplemented with 10% v/v FCS and 50 μg/ml penicillin/streptomycin. Programmed necrosis was induced by addition of human recombinant TRAIL (Super*Killer*TRAIL™, Enzo, Lausen, Germany) or highly purified human recombinant TNF (BASF Bioresearch, Ludwigshafen, Germany), in combination with benzyloxycarbonyl-Val-Ala-Asp(OMe)-fluoromethylketone (zVAD-fmk; Bachem, Heidelberg, Germany) and cycloheximide (CHX; Sigma-Aldrich). In experiments with necrostatin-1, cells were preincubated with 50 μM necrostatin-1 for 2 h before addition of TRAIL/zVAD/CHX or TNF/zVAD/CHX.

### Cytotoxicity assays, viability assays

For flow cytometric analysis of cell death (i.e. loss of membrane integrity), cells were seeded onto 12-well plates at 70% confluence. After treatment, adherent and detached cells were collected, followed by one washing step in PBS/5 mM EDTA. The cells were resuspended in PBS/5 mM EDTA containing 2 *μ*g/ml propidium iodide (PI), and analyzed in a FACSCalibur flow cytometer (BD Biosciences, San Diego, CA, USA) at red fluorescence. Alternatively (when measuring ceramide levels), loss of membrane integrity was determined by trypan blue staining. For this, cells were collected and resuspended in PBS. An aliquot of the cell suspension was added to the same volume of 0.4% v/v trypan blue staining solution (Life Technologies) and applied onto a Neubauer counting chamber. Live cells with an intact cell membrane did not absorb trypan blue and were scored separately from dead (blue) cells. For determination of cell viability by crystal violet staining, cells were seeded in flat-bottom 96-well plates. After stimulation, adherent cells were washed twice with PBS and incubated for 10 min at 37°C in 50 *μ*l of staining solution (0.5% w/v crystal violet, 4% w/v formaldehyde, 30% v/v ethanol, and 0.17% w/v NaCl). The staining solution was washed away with tap water and cells were dried for 1 h at 50°C. Stained cell were dissolved in 33% v/v acetic acid and the absorbance of the staining was measured at 570 nm in a microplate reader (Tecan, Crailsheim, Germany). Suspension cells were alternatively analyzed by metabolic activity measurements with the XTT cell proliferation kit II (Roche, Mannheim, Germany). The intracellular ATP content of cells was determined with the Cell Titer Glo Assay Kit (Promega, Mannheim, Germany) following the instructions of the manufacturer.

### Flow cytometric detection of TRAIL receptors 1 and 2 (TRAIL-R1 and TRAIL-R2)

For detection of cell-surface expression of TRAIL receptors, a total of 1.5 × 10^5^ detached cells were incubated with anti-TRAIL-R1 or anti-TRAIL-R2 mouse monoclonal antibodies (Alexis) in PBS/1% w/v BSA for 1 h at 4°C, washed twice in PBS/1% w/v BSA and incubated with anti-mouse biotin-conjugated secondary antibodies for additional 1 h at 4°C. After two washing steps, cells were incubated with phycoerythrin-conjugated streptavidin for further 15 min at 4°C, washed twice and re-suspended in 150 *μ*l PBS/1% w/v paraformaldehyde and analyzed in a FACSCalibur flow cytometer. Controls were incubated with appropriate isotype matched antibodies and labeled with the corresponding secondary antibodies.

### Western blot

Whole cell lysates were prepared with TNE lysis buffer (50 mM Tris pH 8.0, 150 mM NaCl, 1% v/v NP-40, 3 mM EDTA, supplemented with Complete protease inhibitor mixture (Roche)). The protein lysates were separated by SDS-PAGE, transferred onto nitrocellulose membranes and reactive proteins were detected with antibodies for RIPK1 (BD Biosciences), RIPK3 (Abnova, Heidelberg, Germany), MLKL or actin (Sigma-Aldrich) via chemiluminescence (Lumiglo, Cell Signaling, Danvers, MA, USA). RIPK3 expression was quantified using the program ImageJ (Wayne Rasband, National Institutes of Health, Bethesda, MD, USA). To compare expression levels of RIPK1 and RIPK3 in tumor cell lines, identical amounts of protein (20 μg) were loaded, using lysates from PancTu-I cells as a standard on each gel, and identical exposure times were taken to allow a direct comparison of expression levels. For the quantitative analysis of the relationship between the levels of RIPK3 and the specific sensitivity of the respective tumor cell line to TRAIL/zVAD/CHX-induced programmed necrosis, values for RIPK3 expression were normalized between the gels and calculated relative to CCRF-CEM cells. In all Western blots, detection of actin served as a loading control.

### RNA interference

The predesigned siRNA specific for human RIPK3 (ID # s21741), human MLKL (ID # s47087) as well as the negative control siRNA (ID # AM4611) were obtained from Life Technologies. A second, distinct siRNA specific for human RIPK3 (siGENOME human RIPK3, D-003534-01) was obtained from Thermo Scientific, Schwerte, Germany. U-937 cells were transfected with 150 pmol siRNA by Amaxa nucleofection (Lonza, Cologne, Germany), using solution V and program X-001. HT-29 cells were transfected with 20 pmol siRNA and siPortAmine transfection reagent (Life Technologies).

### Ceramide quantification

Lipids were extracted according to the method of Bligh and Dyer [18] and separated by high-performance thin layer chromatography (TLC) as described [3]. After charring, thin layer chromatography plates were scanned and analyzed using the Molecular Dynamic Personal Densitometer SI Scanner control software (GE Healthcare, Munich, Germany).

### Clonogenic survival assays

Assays for clonogenic survival of cells were essentially carried out as described by Franken and coworkers [19]. Briefly, following treatment, 1,000 viable cells (as determined by trypan blue staining) were plated into six-well plates in complete medium without zVAD-fmk, CHX, TRAIL or TNF, cultured for 7 days at 37°C and stained with crystal violet as described above under “viability assays”, except that all steps subsequent to washing with tap water were omitted. Non-adherent U-937 cells were alternatively analyzed for their ability to form colonies in soft agarose by overlaying them onto 2 ml of 0.4% w/v Sea Plaque agarose (Cambrex, East Rutherford, NJ, USA) on top of 3 ml of a 1% w/v peqGOLD agarose underlayer (PeqLab, Erlangen, Germany), both in complete medium. After incubation for 7 days at 37°C, U-937 cells were stained with 1 ml of 3-[4,5-dimethylthiazol-2yl]-2,5-diphenylterazolium bromide (MTT, Sigma, 2.5 mg/ml in PBS) for 2 h at 37°C to allow metabolization of MTT to blue MTT-formazan. Colony formation (>10 cells) was determined from pictures taken with a Lumix DMC-FS10 digital camera (Panasonic, Wiesbaden, Germany).

### Statistical analysis

For all figures, representative data from one out of at least two or more experiments with similar results are shown (n ≥ 2) and error bars indicate the standard deviations (SD) from at least triplicate determinations (n ≥ 3). *P* values were calculated using Student’s t-test. Statistical significance is denoted by *P < 0.05, **P < 0.01, ***P < 0.001.

## Results

### Sensitivity of human tumor cell lines to TRAIL/zVAD/CHX- and TNF/zVAD/CHX-induced programmed necrosis

**Figure 1:**
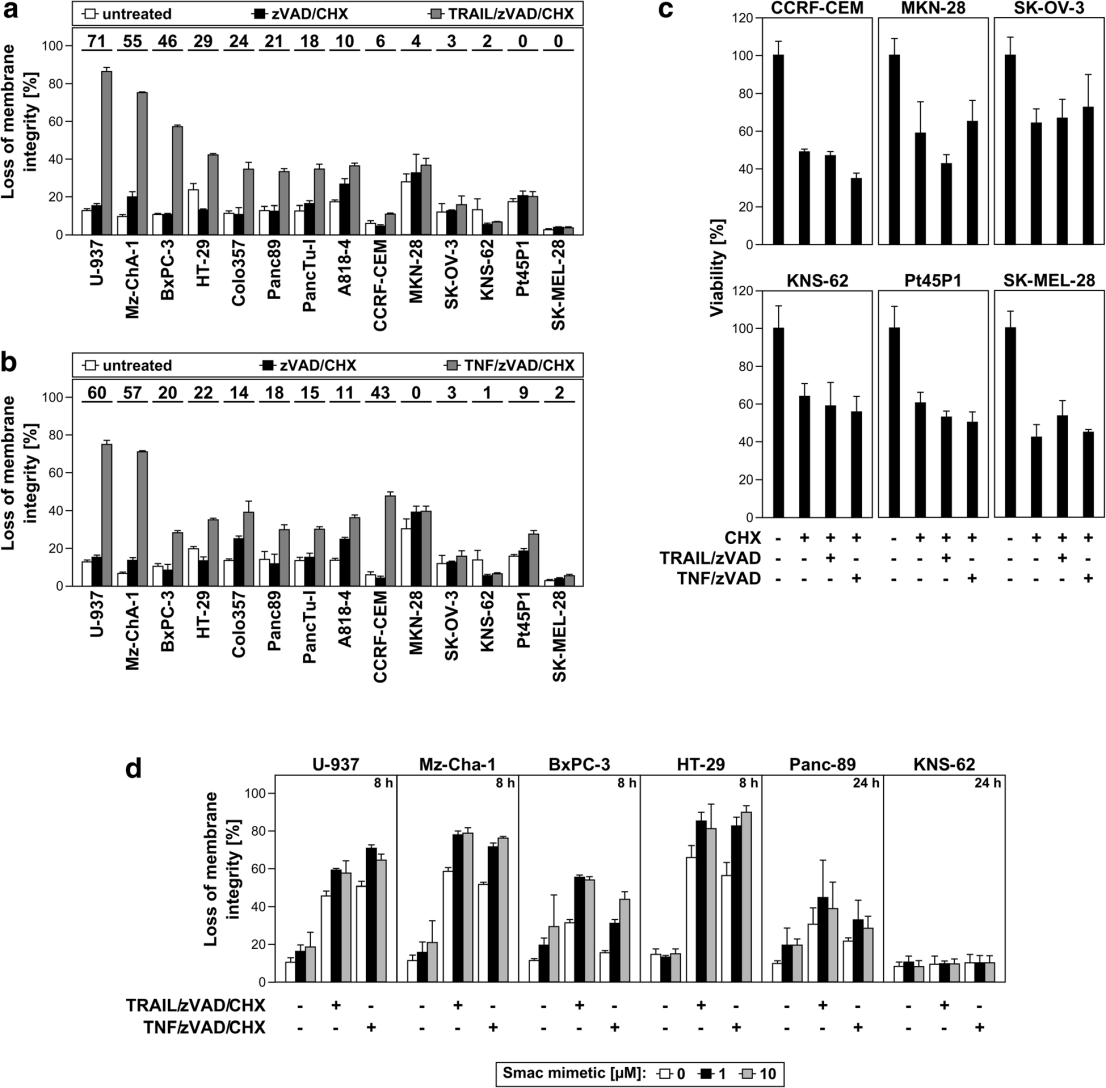
**Induction of programmed necrosis by TRAIL/zVAD/CHX and TNF/zVAD/CHX in human tumor cell lines.** Cells were treated with 100 ng/ml of **(a)** TRAIL or **(b)** TNF in combination with 50 μM zVAD-fmk and non-toxic concentrations of CHX (U-937 0.1 μg/ml; Mz-ChA-1 2 μg/ml; BxPC-3 1 μg/ml; HT-29 5 μg/ml; Colo357 5 μg/ml; Panc89 1 μg/ml; PancTu-I 10 μg/ml; A818-4 10 μg/ml; CCRF-CEM 0.0625 μg/ml; MKN-28 5 μg/ml; SK-OV-3 1 μg/ml; KNS-62 5 μg/ml; Pt45P1 0.1 μg/ml; SK-MEL-28 10 μg/ml). After 24 h, loss of membrane integrity was measured as a marker for programmed necrosis by flow cytometric detection of PI-positive cells. Values above the respective columns represent the specific percentage of programmed necrosis (stimulus-induced minus zVAD/CHX-induced programmed necrosis), spontaneous cell death in untreated cells is shown for comparison. **(c)** Cells were treated with their respective LD_50_ concentrations of CHX alone (CCRF-CEM 60 μg/ml; MKN-28 1000 μg/ml; SK-OV-3 500 μg/ml; KNS-62 300 μg/ml; Pt45P1 150 μg/ml; SK-MEL-28 625 μg/ml) or in combination with 100 ng/ml TRAIL or TNF and 50 μM zVAD-fmk. After 24 h, viability was determined by crystal violet staining (for the adherent cell lines MKN-28, SK-OV-3, KNS-62, Pt45P1 and SK-MEL-28) or XTT assay analysis (for the suspension cell line CCRF-CEM). **(d)** Cells were left untreated or stimulated with TRAIL/zVAD/CHX or TNF/zVAD/CHX as in Figure 1a and b in the presence of the indicated concentrations of the Smac mimetic birinapant. After 8 or 24 h of stimulation, programmed necrosis was analyzed by flow cytometric analysis of PI-positive cells.

We initially characterized the above human cancer cell lines with regard to their sensitivity to TRAIL-induced programmed necrosis, utilizing TNF-elicited programmed necrosis as an established control in this and subsequent experiments. Since treatment with TRAIL normally activates caspase-dependent apoptosis (which would obstruct the analysis of programmed necrosis), we actively inhibited caspases/apoptosis by addition of the broad-spectrum caspase inhibitor zVAD-fmk. This treatment is not only experimentally required to suppress apoptosis, but in addition potentiates programmed necrosis by inhibiting caspase-8, which acts as a negative regulator of programmed necrosis [20] and which otherwise would prevent the induction of programmed necrosis by TRAIL. Furthermore, all cells were additionally treated with non-toxic concentrations of the protein biosynthesis inhibitor cycloheximide (CHX) that we had previously found to sensitize for programmed necrosis. As depicted in Figure 1a, treatment with TRAIL/zVAD/CHX induced programmed necrosis in eight out of 14 tested tumor cell lines. The tumor cell lines U-937, Mz-ChA-1, BxPC-3 and HT-29 exhibited the highest sensitivity, followed by Colo357, Panc89, PancTu-I and A818-4 cells. The remaining cell lines, i.e. CCRF-CEM, MKN-28, SK-OV-3, KNS-62, Pt45P1, and SK-MEL-28 displayed only a marginal or no response to treatment with TRAIL/zVAD/CHX. We obtained essentially the same results in control assays when we induced programmed necrosis with TNF/zVAD/CHX (Figure 1b). As the only exception, CCRF-CEM cells were resistant to TRAIL/zVAD/CHX- but clearly sensitive to TNF/zVAD/CHX-induced programmed necrosis.

In the course of the above experiments, the issue arose whether cell death under the above conditions occurred exclusively by programmed necrosis or whether the combination of TRAIL/zVAD or TNF/zVAD with other cytotoxic agents such as CHX might still result in a net increase in caspase activity and thus in residual apoptosis. Arguing against this assumption, we have previously shown in several studies that no features of apoptosis are detectable in the presence of 20 or 50 μM zVAD-fmk for both TRAIL(/CHX)- and TNF(/CHX)-induced cell death in multiple cell systems. In particular, neither caspase-8 nor caspase-3 activity was detectable in our previous studies, dying cells displayed a necrotic nuclear and cellular morphology, cell death was dependent on RIPK1 and could be blocked by the RIPK1 inhibitor necrostatin-1, no early release of phosphatidylserine or early loss of ∆Ψ_m_ occurred, and the DNA repair enzyme PARP-1 was not cleaved by activated caspase-3 to its apoptotic signature 89-kDa fragment [3,7,17,21]. Moreover, in control experiments that we additionally performed for this study, the tumor cell lines U-937 and HT-29 did not display apoptotic membrane blebbing when treated with TRAIL/zVAD/CHX or TNF/zVAD/CHX. Rather, both cell lines displayed an exclusively necrotic cellular morphology, in contrast to the early and massive blebbing in TRAIL/CHX- or TNF/CHX-treated positive controls for apoptosis ((Additional file 1: Figure S1*a* and Additional file 2: Figure S1*b*)). Furthermore, induction of programmed necrosis in U-937 and HT-29 cells for 24 h with TRAIL/zVAD/CHX did not cause an increase of the 89-kDa PARP-1 cleavage fragment that is generated in apoptosis by activated caspase-3. Likewise, no increase in activated, cleaved capase-3 itself was detectable, whereas induction of apoptosis with TRAIL or TRAIL/CHX in positive controls led to a massive accumulation of cleaved PARP-1 and caspase-3 in both cell lines (Additional file 3: Figures S1*c* and S1*d*). Given that caspase-3 acts downstream of all other apoptotic caspases as the central effector caspase of both extrinsic and intrinsic apoptosis, any (even a small) apoptotic caspase activation would ultimately translate into activation of caspase-3 and thus into an accumulation of cleavage fragments of PARP-1 and caspase-3. However, this was only detectable in the positive controls for apoptosis. Altogether, the above results demonstrate that both TRAIL/zVAD/CHX and TNF/zVAD/CHX induce cell death through programmed necrosis but not through caspase-dependent apoptosis.

To investigate whether a partial or lacking susceptibility of tumor cells to programmed necrosis can be enhanced with stronger sensitization (i.e. higher CHX concentrations), the cell lines CCRF-CEM, MKN-28, SK-OV-3, KNS-62, Pt45P1 and SK-MEL-28 were incubated with their respective lethal dose (LD)_50_ concentrations of CHX alone or in combination with zVAD-fmk and TRAIL (or TNF), employing viability assays as an alternative readout. As shown in Figure 1c, the increased concentrations of CHX were clearly toxic for all cell lines (killing approximately 50% of the cells). Nevertheless, this toxicity was not further enhanced by the addition of TRAIL/zVAD or TNF/zVAD, validating our assay conditions and indicating that the observed susceptibility/resistance of the employed tumor cell lines to programmed necrosis is not a matter of insufficient sensitization.

It has been reported that Smac mimetics can act as enhancers of TNF-induced programmed necrosis [22]. To test whether Smac mimetics can also enhance TRAIL-mediated programmed necrosis, we incubated a set of five arbitrarily selected sensitive tumor cell lines (U-937, Mz-Cha-1, BxPC-3, HT-29 and Panc89; resistant KNS-62 cells were included as control) with TRAIL/zVAD/CHX (or TNF/zVAD/CHX) in the presence of the Smac mimetic birinapant. As shown in Figure 1d, the addition of birinapant indeed led to a further enhancement of both TRAIL/zVAD/CHX- and TNF/zVAD/CHX-induced programmed necrosis in all sensitive tumor cell lines, but not in the resistant tumor cell line KNS-62. Notably, a ten-fold increase in the concentration of birinapant did not further enhance programmed necrosis in the sensitive cell lines or overcome resistance in KNS-62 cells. In summary, these results indicate that similar to TNF/zVAD/CHX-induced programmed necrosis, TRAIL/zVAD/CHX-induced programmed necrosis is mediated by molecular mechanisms that are likewise responsive to Smac mimetics. These data are furthermore consistent with a previous study where it was shown that both TNF- and TRAIL-induced programmed necrosis are enhanced in a RIPK3-dependent manner by combining caspase inhibitors with Smac mimetics [22].

### Cell surface expression of TRAIL-R1 and TRAIL-R2

**Figure 2:**
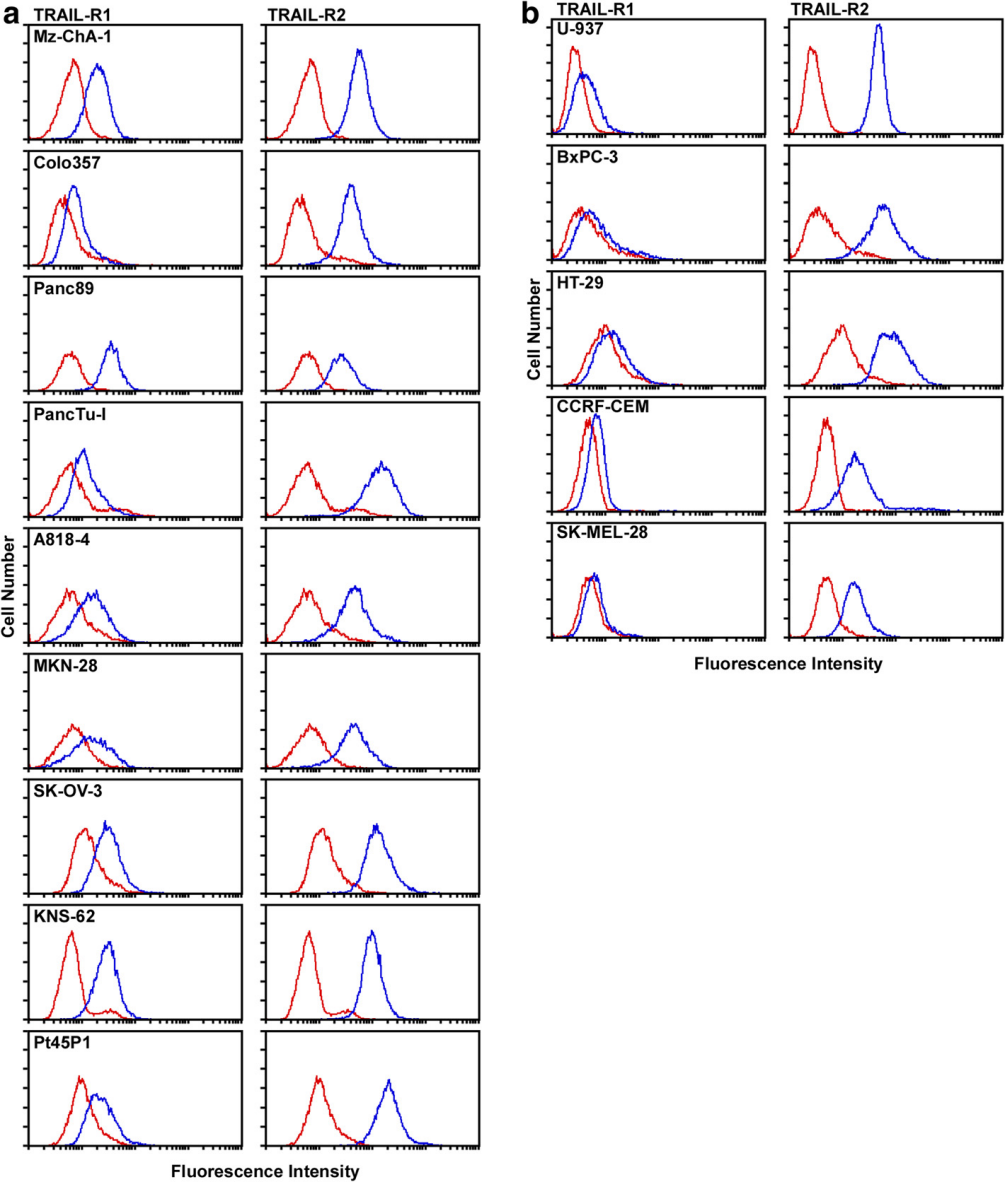
**Flow cytometric analysis of TRAIL-R1 and TRAIL-R2 cell surface expression.** Cell lines clearly expressing both TRAIL-R1 and TRAIL-R2 **(a)** or primarily TRAIL-R2 with low or absent expression of TRAIL-R1 **(b)** are shown. Cells were stained with specific monoclonal antibodies for either TRAIL-R1 or TRAIL-R2 as indicated in the figure (blue) or with isotype matched control antibodies (red), with each curve representing 10,000 counted cells.

The susceptibility/resistance of cells to TRAIL/zVAD/CHX- or TNF/zVAD/CHX-induced programmed necrosis is initially dependent on the expression of the corresponding receptors on the cell surface. We limited our analyses to cell surface expression of TRAIL-R1 and TRAIL-R2 on the tumor cell lines used in this study since TNF-R1 is known to be expressed on the surface of every cell type in the human body except red blood cells [23]. Whereas TRAIL-R1 differentially showed pronounced (Figure 2a) or low to no cell surface expression (Figure 2b), TRAIL-R2 was highly expressed on the surface of all analyzed tumor cell lines (Figures 2a and b). Since Guo and coworkers have shown that TRAIL-R2 can mediate programmed necrosis by itself [10], this indicates that susceptibility or resistance of the investigated tumor cell lines to programmed necrosis is not determined at the level of receptor expression.

### Expression of RIPK3 is a primary determinant for the susceptibility or resistance of tumor cell lines to programmed necrosis

**Figure 3:**
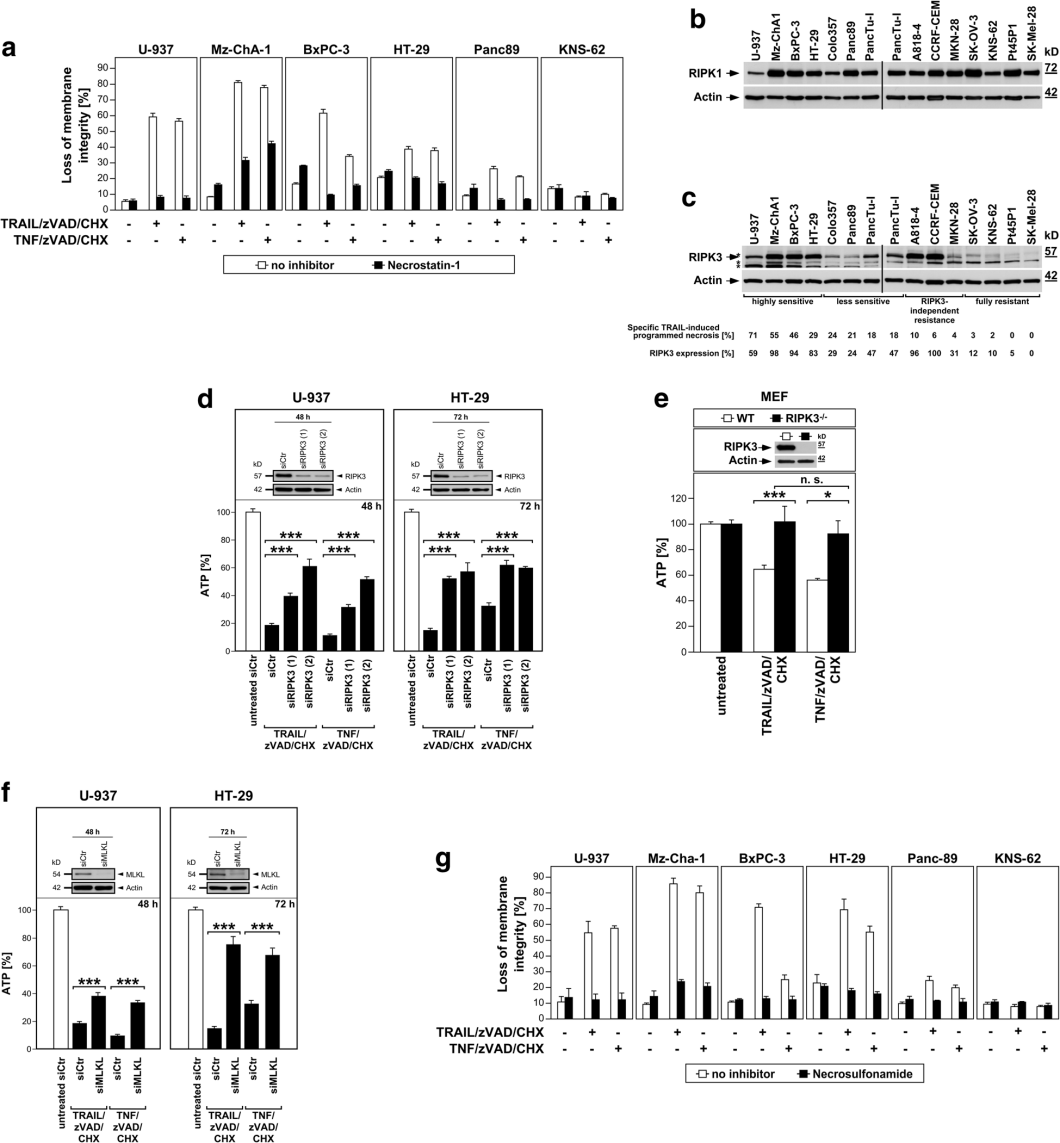
**Role of RIPK1 and RIPK3 as determinants of susceptibility or resistance of tumor cell lines to programmed necrosis. (a)** Cells were analyzed as in Figure 1a and b in the presence or absence of 50 μM necrostatin-1. **(b, c)** Western blots for expression of RIPK1 **(b)** and RIPK3 **(c)**. Asterisks: non-specific bands. RIPK3 corresponds to the lower band of the doublet. A quantitative analysis of the relationship between the levels of RIPK3 and the specific sensitivity of the respective tumor cell line to TRAIL/zVAD/CHX-induced programmed necrosis (values taken from Figure 1a) is shown below. **(d)** U-937 and HT-29 cells were transfected with siRNAs specific for human RIPK3 (two individual siRNAs with distinct target sequences to rule out off-target effects) or a negative control siRNA (siCtr). 48 or 72 h after transfection, cells were left untreated or incubated with TRAIL/zVAD/CHX or TNF/zVAD/CHX as in Figure 1a and b. After 24 h, the decrease of intracellular ATP levels was analyzed as a marker for programmed necrosis. Insets: control Western blots of transfected, untreated cells for downregulation of RIPK3. ***P < 0.001. **(e)** Wild-type (WT) and RIPK3-deficient (RIPK3^−/−^) primary MEF were stimulated with 100 ng/ml of TRAIL or TNF, 20 μM zVAD-fmk and 1 μg/ml CHX for 22 h before ATP levels were measured. Inset: Western blot for RIPK3. *P < 0.05, ***P < 0.001, n. s., not significant. **(f)** As part of the experiment shown in **(d)**, U-937 and HT-29 cells were nucleofected with siRNA specific for MLKL. The data are shown in new panels, but with the same negative control (siCtr) values as in **(d)**. Insets: control Western blots of transfected, untreated cells for downregulation of MLKL. **(g)** Cells were treated and analyzed as in Figure 1a and b in the presence or absence of 1 μM necrosulfonamide.

Programmed necrosis elicited through death receptors critically depends on the protein kinase RIPK1 and the related downstream kinase RIPK3 [24]. We therefore next determined the role of RIPK1 in the investigated tumor cell lines. In line with its essential role in programmed necrosis, pharmacological inhibition of RIPK1 by necrostatin-1 protected the same subset of sensitive tumor cell lines that we had used for analysis in Figure 1d (resistant KNS-62 cells were again included as control) from both TRAIL/zVAD/CHX- and TNF/zVAD/CHX-induced programmed necrosis (Figure 3a). However, Western blots revealed ubiquitous expression of RIPK1 in all 14 cancer cell lines (Figure 3b, (Additional file 4: Figure S2*a*)), demonstrating that their sensitivity or resistance against programmed necrosis is not determined by presence or absence of RIPK1. In contrast to RIPK1, we found a differential expression of RIPK3 that largely correlated with the sensitivity of the tumor cell lines to TRAIL/zVAD/CHX or TNF/zVAD/CHX (Figure 3c, (Additional file 4: Figure S2*b*), Figure 1a and b). Specifically, RIPK3 was clearly expressed in the highly sensitive cell lines U-937, Mz-ChA-1, BxPC-3 and HT-29 but barely detectable in the fully resistant cell lines SK-OV-3, KNS-62, Pt45P1 and SK-Mel28, whereas the less sensitive cell lines Colo357, Panc89 and PancTu-I showed correspondingly reduced levels of RIPK3. Pointing to factors independent from RIPK3 that additionally regulate the resistance of tumor cells against programmed necrosis, MKN-28 cells expressed similar levels of RIPK3 as Colo357 or Panc89 cells but were resistant to both TRAIL/zVAD/CHX and TNF/zVAD/CHX. Likewise, despite strong expression of RIPK3, A818-4 cells responded only poorly to both TRAIL/zVAD/CHX- or TNF/zVAD/CHX-induced programmed necrosis, and CCRF-CEM cells were selectively resistant to TRAIL/zVAD/CHX-induced programmed necrosis (Figure 3c, (Additional file 4: Figure S2*b*), Figure 1a and b). In a complementing experiment, we found that downregulation of RIPK3 by RNA interference in U-937 and HT-29 cells as two sensitive tumor cell lines that express relatively high levels of RIPK3 significantly blocked TRAIL/zVAD/CHX- as well as TNF/zVAD/CHX-induced necrotic killing (Figure 3d). Moreover, RIPK3-deficient but not wildtype MEF were significantly protected from both TRAIL/zVAD/CHX- and TNF/zVAD/CHX-induced programmed necrosis (Figure 3e). In summary, these results suggest expression of RIPK3 as a primary determinant for resistance or susceptibility of the analyzed tumor cells, but also point to secondary factors that additionally confer resistance independent from RIPK3.

It has been reported that phosphorylation of MLKL by RIPK3 is required for RIPK3-dependent programmed necrosis [25,26]. To clarify whether MLKL is also involved in the TRAIL/zVAD/CHX-induced killing of tumor cells, we exemplarily analyzed U-937 and HT-29 cells after downregulation of MLKL. Similar to downregulation of RIPK3, knockdown of MLKL significantly reduced TRAIL/zVAD/CHX- as well as TNF/zVAD/CHX-induced killing in both cell lines (Figure 3f). A comparable protection was conferred by necrosulfonamide, a pharmacological inhibitor of MLKL [27] in the same subset of tumor cell lines that we had used for analysis in Figure 3a (Figure 3g), being furthermore in line with a recent study from Wu and coworkers who found that TRAIL/zVAD/CHX-induced programmed necrosis is compromised considerably in MLKL-deficient mice [27], and in summary identifying MLKL as a mediator not only of TNF/zVAD/CHX-, but also of TRAIL/zVAD/CHX-induced programmed necrosis.

### Ceramide mediates TRAIL/zVAD/CHX- and TNF/zVAD/CHX-induced programmed necrosis in the examined sensitive tumor cell lines

**Figure 4:**
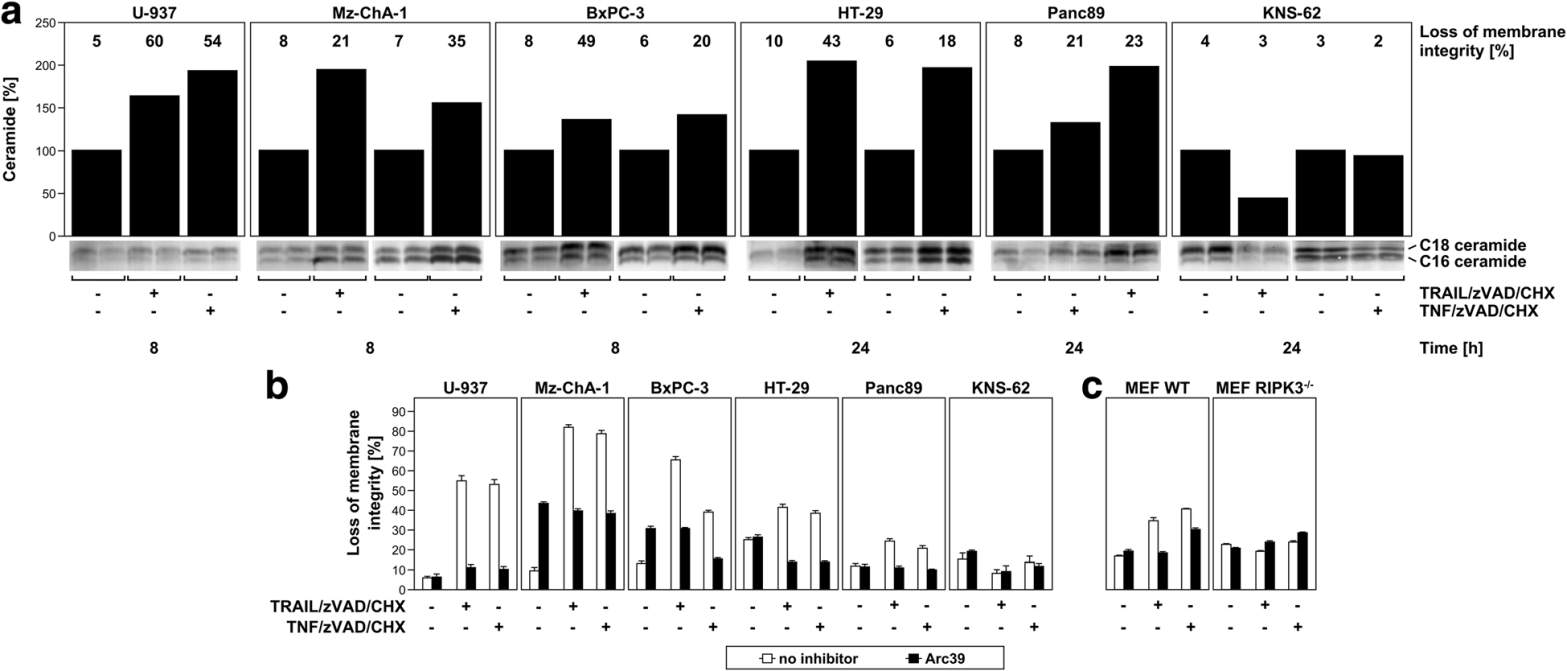
**Ceramide mediates TRAIL/zVAD/CHX- and TNF/zVAD/CHX-induced programmed necrosis in the examined sensitive tumor cell lines. (a)** Cells were left untreated or stimulated with TRAIL/zVAD/CHX or TNF/zVAD/CHX as in Figure 1a and b for the indicated times before intracellular ceramide levels were determined in duplicate. Raw data from the charred TLC plates (C16 and C18 ceramide) are shown below the bar graphs. Loss of membrane integrity as a marker for programmed necrosis was determined in parallel by trypan blue staining and is shown above the respective bars. **(b)** Cells were left untreated or preincubated with 10 μM Arc39 for 2 h before addition of TRAIL/zVAD/CHX or TNF/zVAD/CHX as in Figure 1a and b. After 24 h of stimulation, programmed necrosis was analyzed by flow cytometric analysis of PI-positive cells. **(c)** Wild-type (WT) and RIPK3-deficient (RIPK3^−/−^) primary MEF were left untreated or preincubated with 10 μM Arc39 for 2 h with subsequent addition or not of 100 ng/ml of TRAIL or TNF in combination with 20 μM zVAD-fmk and 1 μg/ml CHX. After 16 h, programmed necrosis was analyzed by flow cytometric analysis of PI-positive cells.

In a previous study, we had identified ceramide generated by the lipase A-SMase as an important mediator of programmed necrosis acting downstream of RIPK1 [3]. However, these studies were performed with common laboratory cell lines, and information on the impact of ceramide as an inducer of programmed necrosis in clinically more relevant tumor cell systems is currently unavailable. Therefore, we studied the intracellular accumulation of ceramide in the same subset of tumor cell lines that we had used for analysis in Figure 3a. As shown in Figure 4a, all five sensitive tumor cell lines but not the resistant cell line KNS-62 displayed a clear accumulation of intracellular ceramide after induction of programmed necrosis by TRAIL/zVAD/CHX or TNF/zVAD/CHX. Moreover, Arc39, a potent and specific inhibitor of A-SMase [11,12] clearly inhibited programmed necrosis in all five sensitive cancer cell lines (Figure 4b), substantiating the previously established role of ceramide as a key element of death receptor-induced programmed necrosis also for the examined tumor cell lines. With regard to the relationship between ceramide signaling and RIPK3 signaling, treatment of primary wildtype MEF with Arc39 likewise protected from TRAIL/zVAD/CHX- and TNF/zVAD/CHX-induced programmed necrosis (Figure 4c), as did the deletion of RIPK3 in primary RIPK3-deficient MEF (Figure 4c, Figure 3e). However, RIPK3-deficient MEF were not further protected by Arc39 (Figure 4c), suggesting that ceramide generated by A-SMase acts downstream of RIPK3 as part of the same signaling pathway.

### Induction of programmed necrosis reduces the clonogenic survival of tumor cells

**Figure 5:**
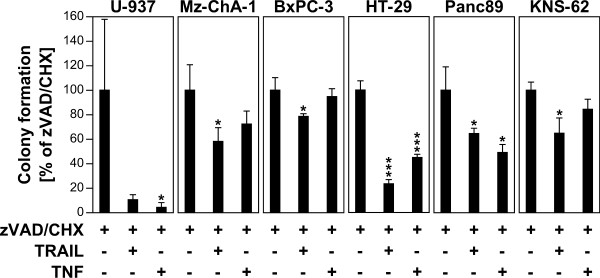
**Impact of TRAIL/zVAD/CHX- and TNF/zVAD/CHX-induced programmed necrosis on clonogenic survival.** Cells were treated with zVAD/CHX, TRAIL/zVAD/CHX or TNF/zVAD/CHX for 24 h as in Figure 1a and b. Subsequently, their ability to form colonies was analyzed by staining with crystal violet (or by soft agarose cloning for the non-adherent cell line U-937) after 7 days. Treatment with zVAD/CHX (negative control) served as reference for calculation of colony formation as well as of *P* values. *P < 0.05, ***P < 0.001.

To determine whether induction of programmed necrosis is a viable strategy to block the capacity of tumor cells for unlimited proliferation, we next investigated clonogenic survival employing the tumor cell lines analyzed in Figures 3a and 4b. As shown in Figure 5, treatment with TRAIL/zVAD/CHX reduced clonogenic survival with statistical significance in four out of five sensitive cell lines (U-937 cells were only slightly above the significance threshold of 0.05 with *P* = 0.059), and even in the control cell line KNS-62 which had shown resistance to TRAIL/zVAD/CHX-induced programmed necrosis in cytotoxicity/viability assays (Figure 1a-c). Almost identical, a reduction of clonogenicity was detectable in five out of the six tested tumor cell lines after treatment with TNF/zVAD/CHX, with three cell lines showing a statistically significant reduction (KNS-62 cells were only slightly above the significance threshold with *P* = 0.057). In summary, these data confirm that induction of programmed necrosis can reduce the proliferative potential and thus the clonogenicity of tumor cells.

### TRAIL/zVAD/CHX-induced programmed necrosis synergizes with chemotherapy in the elimination of tumor cells

**Figure 6:**
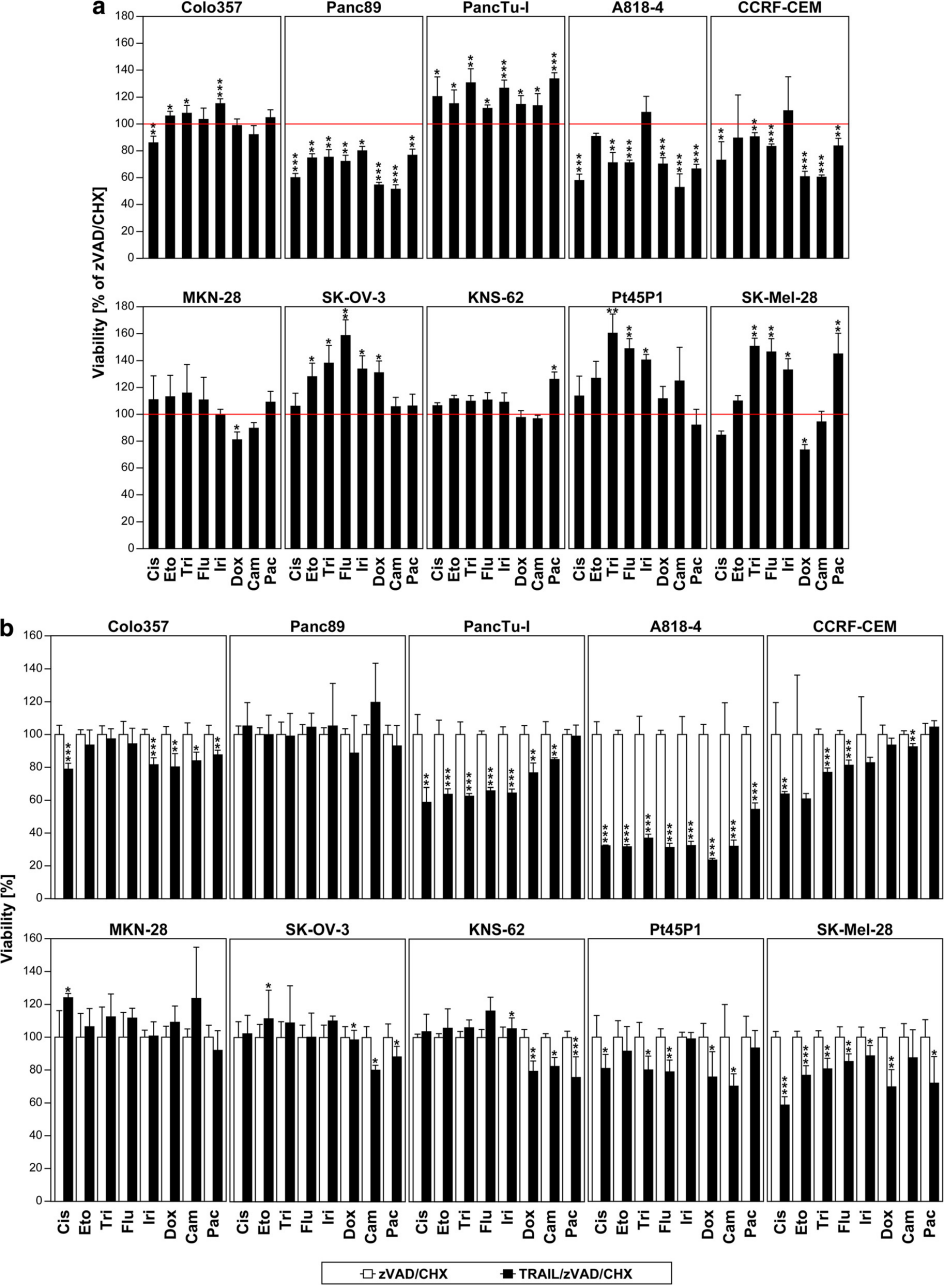
**TRAIL/zVAD/CHX-induced programmed necrosis synergizes with chemotherapy in the elimination of tumor cells. (a)** Cells were treated with zVAD/CHX as in Figure 1a with additional application of cisplatin (Cis, 50 μM), etoposide (Eto, 20 μg/ml), trichostatin A (Tri, 100 ng/ml), 5-fluorouracil (Flu, 50 μg/ml), irinotecan (Iri, 1 μM), doxorubicin (Dox, 5 μg/ml), camptothecin (Cam, 10 μM), or paclitaxel (Pac, 1 μM). After 24 h of incubation, viability was measured with the XTT detection kit (for non-adherent CCRF-CEM cells) or by crystal violet staining (for all other cell lines) and is shown relative to zVAD/CHX-treated cells. Treatment with zVAD/CHX (control) served as reference for the calculation of *P* values. **(b)** In the same experiment, cells were additionally treated with TRAIL/zVAD/CHX as in Figure 1a with addition of chemotherapeutics as in **(a)**. Here, viability is shown relative to cells treated with zVAD/CHX in combination with the respective chemotherapeutic agent, which also served as reference for the calculation of *P* values. *P < 0.05, **P < 0.01, ***P < 0.001.

For the apoptotic elimination of tumor cells, combination therapies of TRAIL and chemotherapeutic agents have been comprehensively investigated [9]. In contrast, a corresponding synergism of chemotherapeutic agents and TRAIL-induced programmed necrosis has hardly been examined. To address this issue, we analyzed all but the four most TRAIL/zVAD/CHX-sensitive tumor cell lines in viability assays after treatment with the chemotherapeutic agents cisplatin, etoposide, trichostatin A, 5-fluorouracil, irinotecan, doxorubicin, camptothecin, or paclitaxel in the presence of zVAD/CHX. Although some cell lines (Colo357, PancTuI, MKN-28, SK-OV-3, KNS-62, Pt45P1, SK-Mel-28) were largely resistant or even responded with increased viability, a combination of chemotherapeutic agents and zVAD/CHX alone already induced cytotoxicity in other tumor cell lines (Panc89, A818-4, CCRF-CEM), demonstrating that chemotherapeutic agents can kill tumor cells not only by apoptosis, but also by programmed necrosis (Figure 6a). Even more encouraging, the addition of TRAIL significantly enhanced the cytotoxic effect of chemotherapeutics in eight out of 10 tumor cell lines and in 41 out of a total of 80 chemotherapeutic/TRAIL/zVAD/CHX combinations (Figure 6b). Notably, the combined induction of programmed necrosis by chemotherapeutic agents and TRAIL/zVAD/CHX led to a broad and statistically significant reduction of viability in three cell lines which had shown resistance to chemotherapeutic agents and zVAD/CHX alone (Colo357, PancTu-I, Pt45P1), and likewise (but more limited to specific chemotherapeutics) in two further cell lines (SK-OV-3, KNS-62). Finally, the cytotoxic response of the cell lines A818-4, CCRF-CEM and SK-Mel-28 which had proven sensitive already to chemotherapeutic agents and zVAD/CHX alone was clearly further enhanced by the addition of TRAIL (Figures 6a and b), in summary demonstrating that TRAIL/zVAD/CHX synergizes with chemotherapeutic agents in the induction of programmed necrosis, and that this treatment may represent a promising new option for the development of future combination therapies.

## Discussion and conclusion

In this study, we have investigated whether induction of TRAIL/zVAD/CHX-induced programmed necrosis represents a viable strategy for the elimination of tumor cells. Necrosis has long been regarded as an accidental, non-physiologic form of cell death, whereas caspase-dependent apoptosis was considered to be the only form of programmed and thus physiologically occurring cell death. This view has however been challenged by numerous studies which have provided evidence for the existence of programmed forms of necrosis that do not depend on caspases but nevertheless follow defined molecular steps [20]. While caspase-dependent apoptosis is the major pathway leading to PCD, programmed necrosis can act as a backup system when the apoptotic machinery fails or is inactivated (e.g. by mutations in apoptosis-resistant cancer cells) [5,28]. It has been shown that programmed necrosis exerts critical functions in multiple patho-physiological settings, e.g. the regulation of bone growth, ovulation, negative selection of lymphocytes [28], pancreatitis [22,29], epilepsy, ischemia–reperfusion injury, Parkinson’s, Huntington’s and Alzheimer's disease, and cell destruction by Salmonella, Shigella, HIV and vaccinia virus [4,28,30,31]. In contrast to apoptosis, a comprehensive picture of the signaling pathways of programmed necrosis is not yet available. In the most extensively studied model, TNF-R1 elicits programmed necrosis via activation of RIPK1 and RIPK3, a step which is stimulated by the deubiquitinase CYLD and the deacetylase SIRT2, but negatively regulated by the proteins FADD, FLIP, caspase-8 and members of the cIAP family. Downstream of RIPK3, the proteins MLKL and PGAM5 contribute to programmed necrosis by promoting mitochondrial fragmentation [20]. We have previously demonstrated that ceramide acts as an additional key molecule in death receptor-mediated programmed necrosis [3,6,7]. Furthermore, enzymes of the energy metabolism, the Bcl-2-family member Bmf and production of reactive oxygen species have been implicated as additional factors in programmed necrosis [4].

The capacity to elicit programmed necrosis appears to be an intrinsic feature of death receptors and has been reported not only for TNF-R1 [2,3,6], but also for Fas/CD95 [4] and ectodermal dysplasia receptor [32]. Independently, we and others have demonstrated the ability to trigger programmed necrosis for human and murine TRAIL receptors [7,10]. In contrast to programmed necrosis, the efficacy of TRAIL in the apoptotic elimination of tumor cells has been extensively demonstrated in clinical trials employing mono- or combination therapies [9]. Consistent with the finding that TRAIL elicits apoptosis selectively in tumor but not primary cells, TRAIL was well tolerated in preclinical models at serum concentrations that were shown to be effective against cancer cells, as were agonistic TRAIL receptor antibodies applied to patients in clinical trials using mono- or combination therapies [9]. Nevertheless, intrinsic resistance against TRAIL-induced apoptosis, even when combined with chemo- or radiotherapy, limit the therapeutic success and necessitate the search for additional, yet unexplored options for the treatment of patients.

As such a potential option, the induction of programmed necrosis by TRAIL has however been investigated only in a very limited number of studies. Our own study presented here provides strong evidence for the suitability of TRAIL/zVAD/CHX-induced programmed necrosis as a tool to eliminate tumor cells from a wide range of sources. Raising the expectation that TRAIL/zVAD/CHX-induced programmed necrosis may be even more effective under conditions that more closely resemble the *in vivo* situation than mere cell culture, it clearly interfered with the capacity of all tested tumor cell lines for unlimited proliferation in clonogenic survival assays (even in a tumor cell line that had shown resistance in conventional cytotoxicity/viability assays). Furthermore, our data demonstrate that cisplatin, etoposide, trichostatin A, 5-fluorouracil, irinotecan, doxorubicin, camptothecin and paclitaxel can exert cytotoxicity not only via apoptosis, but also via programmed necrosis. Providing additional encouragement for the development of future combination therapies, TRAIL/zVAD/CHX-induced programmed necrosis synergized with chemotherapeutic agents and enhanced the cytotoxic response in eight out of 10 tested tumor cell lines as well as 41 out of 80 chemotherapeutic/TRAIL/zVAD/CHX combinations. With regard to potential predictive markers, our results identify expression of RIPK3 as a primary determinant of susceptibility or resistance of tumor cells to TRAIL/zVAD/CHX-induced programmed necrosis. However, our data also show that in future screenings, it should be kept in mind that secondary factors may additionally confer resistance downstream or independent from RIPK3. Finally, our study has confirmed and extended the role of ceramide as one of the key mediators of programmed necrosis downstream of RIPK1 and RIPK3 to the clinically more relevant tumor cell systems investigated here, with the A-SMase inhibitor Arc39 additionally validating A-SMase (rather than neutral sphingomyelinase or ceramide synthase) as the main enzyme responsible for ceramide generation. Our findings are not only fully consistent with our previous data from the initially studied laboratory cell lines [3,6,7], but may also prove valuable for a future manipulation of intracellular ceramide levels to induce programmed necrosis in tumor therapy.

As pointed out above, only very few other studies have focused on the induction of programmed necrosis by TRAIL. One of those studies has recently reported that TRAIL induces necroptosis (i.e. a subset of programmed necrosis depending on RIPK1/RIPK3 [20]) in the tumor cell lines HT-29 (which was also used in this study) and Hep G2 [33], at first glance consistent with our results. However, unlike in our study, necroptosis was only observed under acidified (but not physiologic) conditions. Moreover, the same group had previously reported that in this very system, caspase activity is required for cell death [34], being inconsistent with the molecular mechanisms described for necroptosis [20] and thus suggesting a certain caution when interpreting the results of this study. More encouraging, Hunter and coworkers have reported that TRAIL can kill small cell lung cancer cells by inducing caspase-independent mechanisms of cell death in synergy with paclitaxel [35]. Independently, platinum compounds in combination with TRAIL were found to be effective against breast cancer cells by inducing programmed necrosis (although to a lesser extent) in addition to apoptosis [36]. Finally, Katz and colleagues have described that malignant pleural mesothelioma cells are killed by caspase-independent mechanisms after application of TRAIL in combination with sorafenib, and even find promising evidence of therapeutic efficacy in a xenograft mouse model [37], in summary corroborating our data on the synergistic action of TRAIL/zVAD/CHX and chemotherapy in the programmed necrosis of tumor cell lines.

With regard to a future therapeutic application of TRAIL/zVAD/CHX-induced programmed necrosis, further work is required. At present, it is unknown whether RIPK3-proficient tumor cells can be stimulated to undergo programmed necrosis and thus circumvent apoptosis resistance in patients. For this purpose, strategies for the induction of programmed necrosis (e.g. as used in our study) need to be adapted to the *in vivo* situation. As an example, the sensitizer CHX used here is cytotoxic also to healthy tissue. Therefore, therapies based on the treatment of patients with CHX are not an option. As a possible alternative (and in line with our own data presented in Figure 1d), Smac mimetics can similarly sensitize tumor cells for TRAIL- and TNF-induced programmed necrosis in cell culture models [22]. Yet, their efficacy or toxicity under conditions of programmed necrosis has not been evaluated *in vivo* so far.

Since this study focuses on TRAIL-induced programmed necrosis as a novel approach to eliminate tumor cells, we explicitly want to point out that TRAIL-induced programmed necrosis in principle occurs under the condition that the normal apoptotic pathway is inhibited. It has recently become clear that caspase-8 suppresses programmed necrosis under normal conditions and that it needs to be actively inhibited (e.g. by zVAD-fmk) for programmed necrosis to be executed. Notably, even the basal activity of non-stimulated caspase-8 is already sufficient for the suppression of programmed necrosis [20]. Therefore, the induction of programmed necrosis in apoptosis-resistant cell lines in the absence of caspase inhibitors would only be effective in tumors that carry a mutation that directly inactivates caspase-8. In all other cases (i.e. in cells that harbor apoptosis-inhibiting mutations affecting other proteins) the residual activity of caspase-8 would still be sufficient to suppress programmed necrosis. Most likely, this is the reason why the application of TRAIL alone has so far not been effective against apoptosis-resistant tumors in clinical trials. Therefore, we consider the inhibition of caspase-8 as an essential prerequisite for the successful elimination of tumor cells by TRAIL-induced programmed necrosis. In future treatment regimens this could be most conveniently achieved by combining TRAIL with a caspase inhibitor such as zVAD-fmk. With regard to its physiological and clinical relevance, zVAD-fmk has so far proven to be a non-toxic substance that has no adverse effects and which is well tolerated when administered for prolonged periods of time [3,38,39]. However, although TRAIL and zVAD-fmk by themselves have not shown toxicity *in vivo*[9,40], it must be clarified whether their joint application (and additionally in combination with chemotherapeutic agents) is equally non-toxic *in vivo*.

As another topic to be addressed with regard to future therapies, cells dying by programmed necrosis can release a broad range of damage-associated molecular patterns (DAMPs) which in turn can trigger inflammatory responses. Accordingly, programmed necrosis has been associated with inflammation in several *in vivo* models [20]. Therefore, it will be of high interest to clarify whether the death of tumor cells via programmed necrosis is immunogenic and may thus elicit a highly desirable anticancer immune response that would eliminate residual tumor (stem) cells [4]. Such a beneficial inflammation elicited by tumor cells undergoing TRAIL-induced programmed necrosis could thus contribute to an even more effective treatment for cancer patients in the future.

## Abbreviations

CHX: Cycloheximide; MEF: Mouse embryonic fibroblast; MTT: 3-[4,5-dimethylthiazol-2yl]-2,5-diphenyltetrazolium bromide; PCD: Programmed cell death; PI: Propidium iodide; SD: Standard deviation; siRNA: Small interfering RNA; TLC: Thin layer chromatography; TNF: Tumor necrosis factor; TNF-R1: TNF receptor 1; TRAIL: Tumor necrosis factor-related apoptosis-inducing ligand; TRAIL-R1/2: TRAIL receptor 1/2; zVAD-fmk: Benzyloxycarbonyl-Val-Ala-Asp(OMe)-fluoromethylketone.

## Competing interests

The authors declare that they have no competing interests.

## Authors’ contributions

SV, SP, SS, HK and DA designed research; SV, SP, PD and SWM performed research; SV, SP, SWM, CR, CA, AT, DK, SS, HK and DA analyzed data, SV and DA wrote the paper. All authors read and approved the final manuscript.

## Pre-publication history

The pre-publication history for this paper can be accessed here:

http://www.biomedcentral.com/1471-2407/14/74/prepub

## Supplementary Material

Additional file 1: Figure S1a Treatment with TRAIL/zVAD/CHX or TNF/zVAD/CHX does not elicit caspase-dependent apoptosis in tumor cells. Click here for file

Additional file 2: Figure S1b Treatment with TRAIL/zVAD/CHX or TNF/zVAD/CHX does not elicit caspase-dependent apoptosis in tumor cells. Click here for file

Additional file 3: Figure S1c and **Figure S1** ***d***. Treatment with TRAIL/zVAD/CHX or TNF/zVAD/CHX does not elicit caspase-dependent apoptosis in tumor cells. Click here for file

Additional file 4: Figure S2 Role of RIPK1 and RIPK3 as determinants of susceptibility or resistance of tumor cell lines to programmed necrosis. Click here for file
