# Supplementary figures and images for: TRAIL-induced programmed necrosis as a novel approach to eliminate tumor cells
Source: BMC Cancer. 2014 Feb 7;14:74. doi: 10.1186/1471-2407-14-74 (PMC3927850; doi:10.1186/1471-2407-14-74)

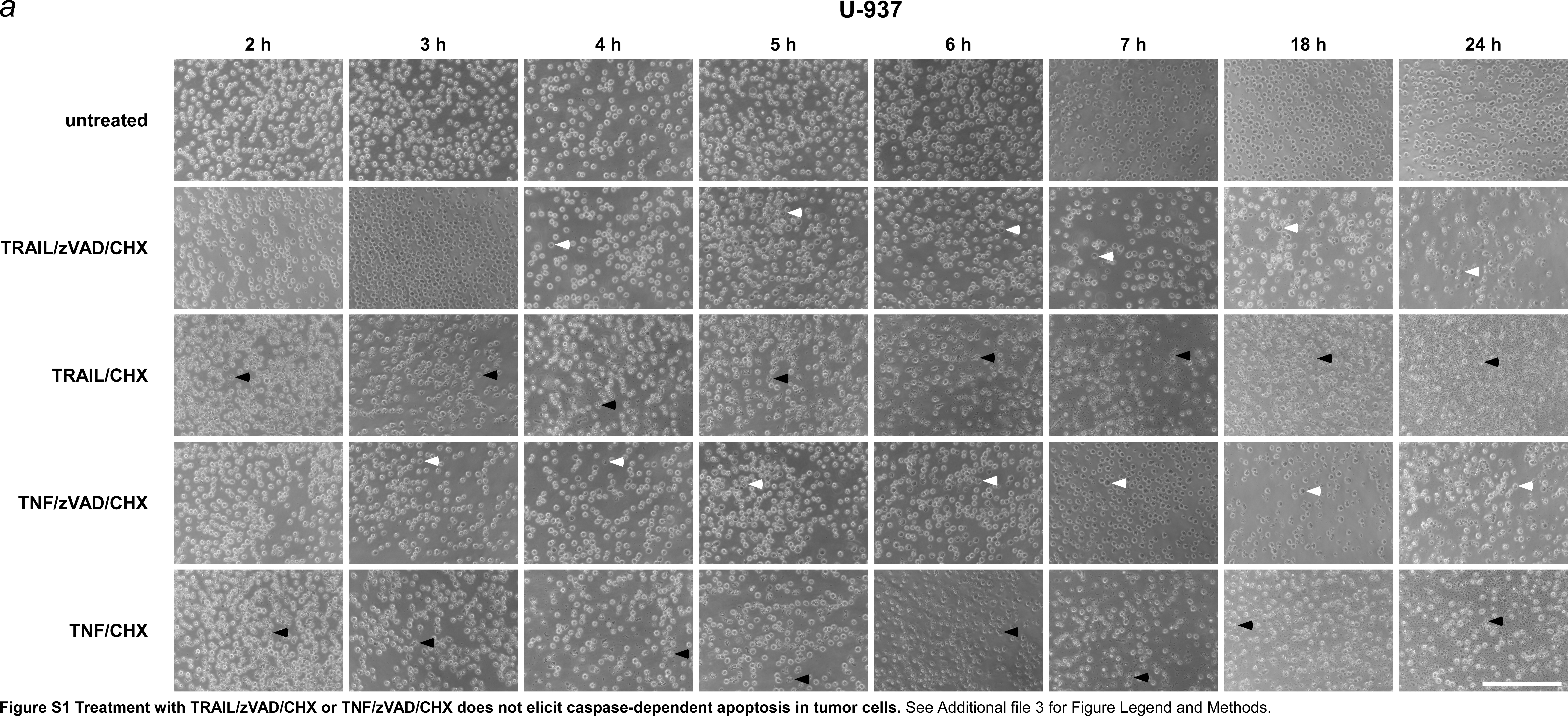

Supplement: Additional file 1: Figure S1a — Treatment with TRAIL/zVAD/CHX or TNF/zVAD/CHX does not elicit caspase-dependent apoptosis in tumor cells. [file 1471-2407-14-74-S1.tif]

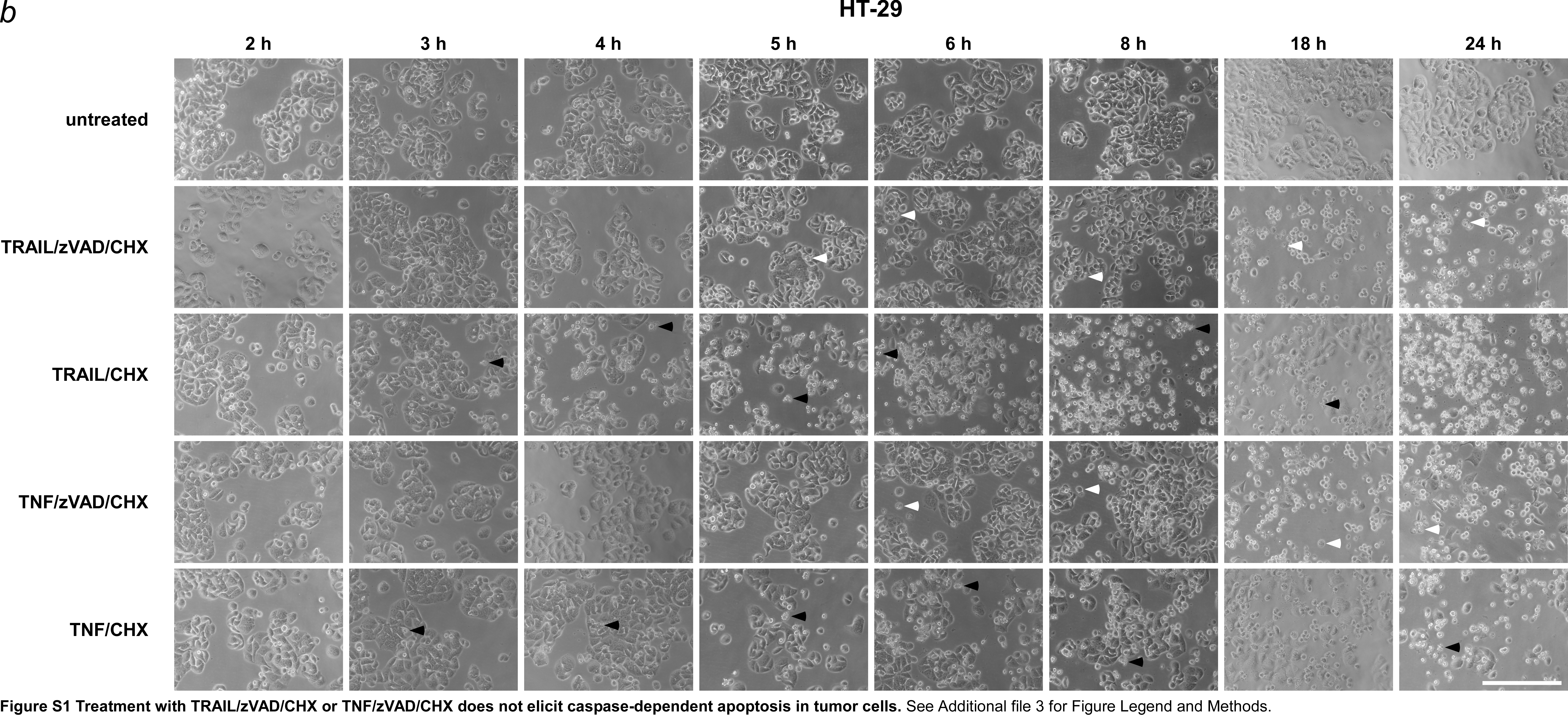

Supplement: Additional file 2: Figure S1b — Treatment with TRAIL/zVAD/CHX or TNF/zVAD/CHX does not elicit caspase-dependent apoptosis in tumor cells. [file 1471-2407-14-74-S2.tif]

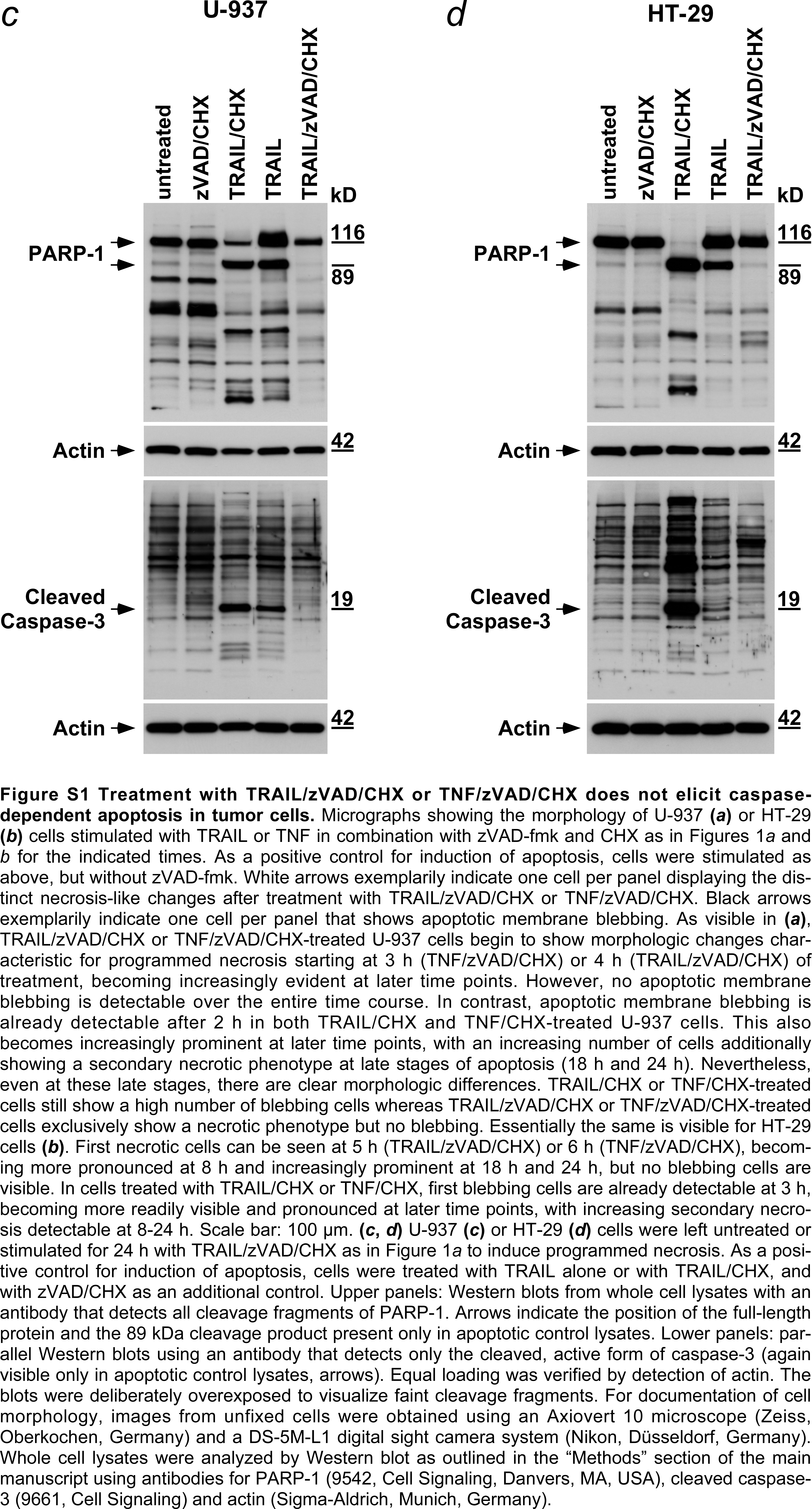

Supplement: Additional file 3: Figure S1c — and Figure S1 d . Treatment with TRAIL/zVAD/CHX or TNF/zVAD/CHX does not elicit caspase-dependent apoptosis in tumor cells. [file 1471-2407-14-74-S3.tif]

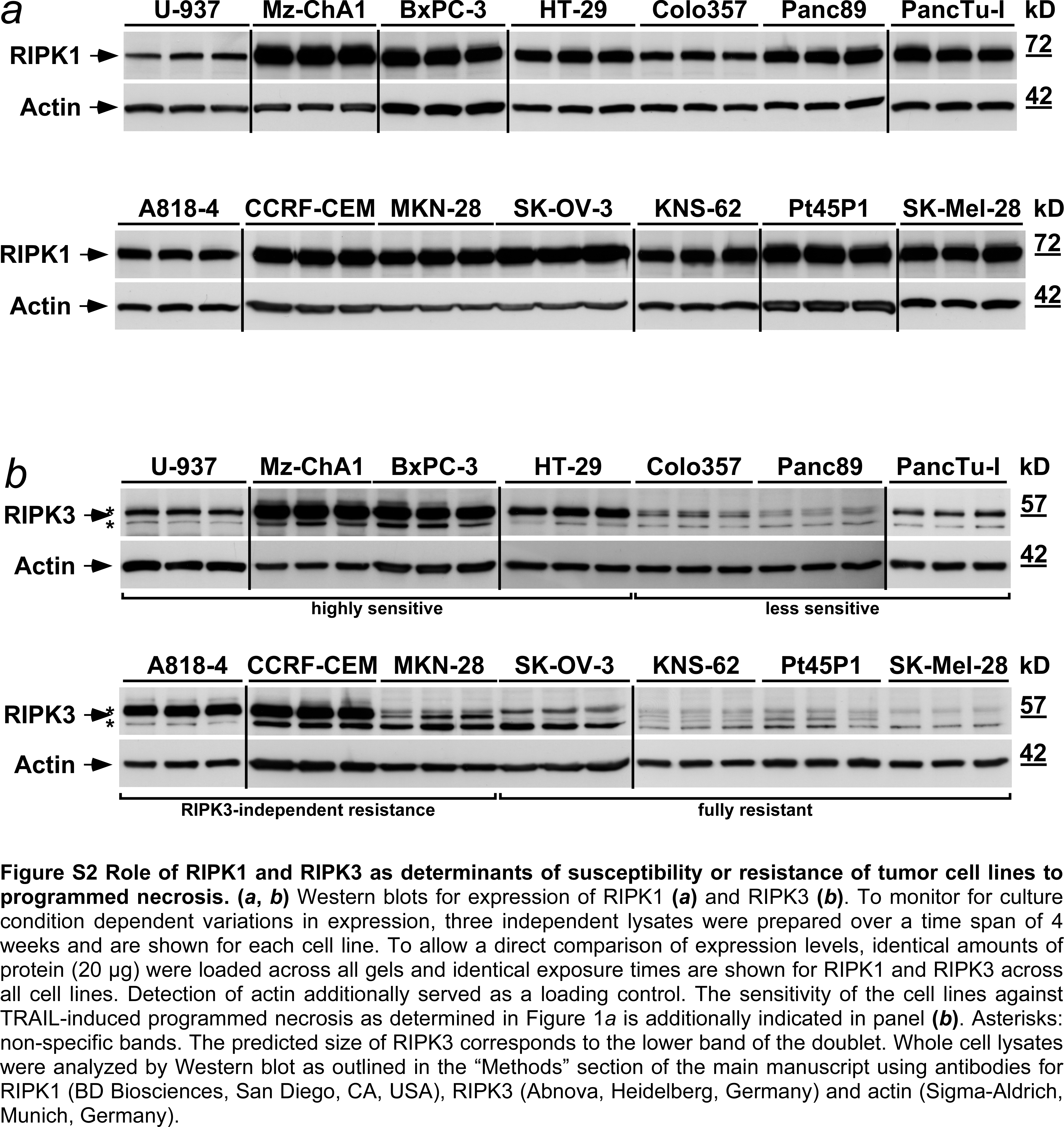

Supplement: Additional file 4: Figure S2 — Role of RIPK1 and RIPK3 as determinants of susceptibility or resistance of tumor cell lines to programmed necrosis. [file 1471-2407-14-74-S4.tif]
